# Identification of degradome components associated with prostate cancer progression by expression analysis of human prostatic tissues

**DOI:** 10.1038/sj.bjc.6602630

**Published:** 2005-05-31

**Authors:** A C P Riddick, C J Shukla, C J Pennington, R Bass, R K Nuttall, A Hogan, K K Sethia, V Ellis, A T Collins, N J Maitland, R Y Ball, D R Edwards

**Affiliations:** 1 Norfolk and Norwich University Hospital NHS Trust, Norwich NR4 7UY, UK; 2 School of Biological Sciences, University of East Anglia, Norwich NR4 7TJ, UK; 3 YCR Cancer Research Unit, Department of Biology, University of York, YO 10 5YW, UK

**Keywords:** matrix metalloproteinases, serine proteases, prostate cancer

## Abstract

Extracellular proteases of the matrix metalloproteinase (MMP) and serine protease families participate in many aspects of tumour growth and metastasis. Using quantitative real-time RT–PCR analysis, we have undertaken a comprehensive survey of the expression of these enzymes and of their natural inhibitors in 44 cases of human prostate cancer and 23 benign prostate specimens. We found increased expression of *MMP10, 15, 24, 25* and *26*, urokinase plasminogen activator-receptor (*uPAR*) and plasminogen activator inhibitor-1 (*PAI1*), and the newly characterised serine proteases *hepsin* and matriptase-1 (*MTSP1*) in malignant tissue compared to benign prostate tissue. In contrast, there was significantly decreased expression of *MMP2* and *MMP23, maspin*, and the protease inhibitors tissue inhibitor of metalloproteinase 3 (*TIMP3*), *TIMP4* and *RECK* (reversion-inducing cysteine-rich protein with Kazal motifs) in the cancer specimens. The expression of *MMP15* and *MMP26* correlated positively with Gleason score, whereas *TIMP3*, *TIMP4* and *RECK* expression correlated negatively with Gleason score. The cellular localisation of the expression of the deregulated genes was evaluated using primary malignant epithelial and stromal cell cultures derived from radical prostatectomy specimens. *MMP10* and *25, hepsin, MTSP1* and *maspin* showed predominantly epithelial expression, whereas *TIMP 3 and 4, RECK, MMP2* and *23, uPAR* and *PAI1* were produced primarily by stromal cells. These data provide the first comprehensive and quantitative analysis of the expression and localisation of MMPs and their inhibitors in human prostate cancer, leading to the identification of several genes involved in proteolysis as potential prognostic indicators, in particular *hepsin, MTSP1, MMP26, PAI1, uPAR, MMP15, TIMP3, TIMP4, maspin* and *RECK*.

Prostate cancer will affect one man in five and is now the leading cause of cancer deaths in Western men (Levi *et al*, 2004). The biological mechanisms underlying its initiation and progression are incompletely understood and there is an urgent need for the identification of robust prognostic markers, so that the most effective treatment can be provided. For example, the optimum treatment of patients with localised prostate cancer is uncertain and the identification of molecular markers that are predictive of increased malignant potential could make an important contribution to therapeutic decision-making.

Degradation of the extracellular matrix (ECM) is integral in tumorigenesis and is mediated by the activity of ECM proteases, including the matrix metalloproteinases (MMPs) and the serine proteases (Sternlicht and Werb, 2001; Egeblad and Werb, 2002). There are 24 members of the human MMP family (Lopez-Otin and Overall, 2002) that have a number of other roles in tumorigenesis, including a direct role in ECM degradation mediating tumour establishment, growth and migration, avoidance of apoptosis, angiogenesis and interaction with the immune system. These effects are achieved in part by the cleavage of growth factors, their receptors, or other growth factor-associated proteins (Egeblad and Werb, 2002). Matrix metalloproteinase activity is regulated at several levels. Activation of most MMPs occurs extracellularly and is mediated by proenzyme cleavage involving other MMPs and serine proteases, in particular the plasminogen activator-plasminogen network (Egeblad and Werb, 2002). An important means of their inhibition is achieved by the binding of the tissue inhibitors of metalloproteases (TIMPs), of which there are four members (Jiang *et al*, 2002; Baker *et al*, 2002). In addition, the recently characterised MMP inhibitor, RECK (reversion inducing cysteine-rich protein with Kazal motifs) can exert effects on the synthesis, activation and activity of several MMPs (Oh *et al*, 2001; Sasahara *et al*, 2002). Members of the serine protease family have been shown to be important in a variety of malignant tumours. In particular, the plasminogen activator system, which mediates cell surface plasmin generation via association of urokinase plasminogen activator (uPA) with its glyco-phosphoinositol-anchored receptor (uPAR), thus leading to degradation of pericellular proteins directly, as well as indirectly through activation of several pro-MMPs (Andreasen *et al*, 2000; Egeblad and Werb, 2002; Ellis, 2003; Freije *et al*, 2003). Moreover, transmembrane serine proteases (e.g. hepsin and matriptase) have also been implicated in tumorigenesis, but their roles are as yet unresolved (Dhanasekaran *et al*, 2001; Stamey *et al*, 2001; Srikantan *et al*, 2002; Chen *et al*, 2003; Galkin *et al*, 2004).

There is considerable evidence supporting the involvement of MMPs and TIMPs in prostate cancer (Pajouh *et al*, 1991; Lokeshwar *et al*, 1993; Boag and Young, 1994; Wood *et al*, 1997; Hashimoto *et al*, 1998; Still *et al*, 2000; Srikantan *et al*, 2002; Trudel *et al*, 2003; Udayakumar *et al*, 2003; Zhao *et al*, 2003). However, research has focused almost entirely on the earlier discovered MMPs (MMP-2, -7 and -9) and TIMP-1 and -2; the more recently discovered MMPs have received little attention (Zhao *et al*, 2003). Similarly, little is known of the potential roles of the transmembrane serine proteases and their inhibitors. However, recent data from microarray profiling studies have linked hepsin with prostate cancer pathogenesis (Dhanasekaran *et al*, 2001; Stamey *et al*, 2001; Ernst *et al*, 2002; Chen *et al*, 2003).

In this study we investigated the expression of the entire MMP and TIMP families, RECK, and selected serine proteases and inhibitors in a large collection of primary malignant and nonmalignant prostate tissues. In the absence of monoclonal antibodies of equivalent specificity and sensitivity, we used quantitative real-time PCR assay (Nuttall *et al*, 2003) to measure mRNA levels. In order to investigate the cellular localisation of these genes, we measured mRNA levels in primary epithelial and stromal cell cultures derived from malignant prostate tissues. These data provide the first detailed descriptions of the stromal *vs* epithelial origin of the expression of the proteases and inhibitors, and indicate that several proteolysis-associated genes may be useful prognostic indicators in prostate cancer.

## MATERIALS AND METHODS

### Clinical samples

Samples of malignant and nonmalignant human prostate tissue were obtained from the Partners in Cancer Research Tissue Bank, held in the Department of Histopathology at the Norfolk & Norwich University Hospital (NNUH). Detailed procedures for obtaining informed patient consent, tissue acquisition, and histopathological and molecular quality control and validation have already been described (Riddick *et al*, 2003). Samples of malignant prostate tissue were collected from patients undergoing radical prostatectomy or channel transurethral resection of the prostate (TURP), and nonmalignant samples were obtained from patients undergoing radical cystoprostatectomy for transitional cell carcinoma of the bladder or TURP for benign prostatic hyperplasia. Gene expression profiling was undertaken in malignant tissue samples from 44 patients with adenocarcinoma of the prostate (four with Gleason scores 5–6, 25 with Gleason score 7, and 15 with Gleason scores 8–10) and in nonmalignant samples from 23 other patients with benign prostatic hyperplasia. The data were further analysed to relate gene expression to increasing Gleason score.

### Primary culture of malignant epithelial and stromal cells from adult, human prostate

Malignant human prostate tissue was obtained, with patient consent, from nine patients (age range 52–68 years) undergoing radical prostatectomy for prostate cancer. One sample was derived from a Gleason grade 6 carcinoma and the remaining specimens from grade 7 tumours. The presence of prostate cancer was confirmed by histological examination of representative fragments. The tissue was prepared as described previously (Collins *et al*, 2001). Briefly, collagenase digestion released epithelial structures (organoids; glands and ducts), which where subsequently separated from the stromal fraction by repeated unit gravity centrifugation. The stromal cells were routinely cultured in RPMI 1640 medium supplemented with 10% foetal calf serum (FCS) and the antibiotics penicillin (100 U ml^−1^; Invitrogen) and streptomycin (100 *μ*g ml^−1^; Invitrogen). Epithelial cultures were grown in keratinocyte serum-free medium (KSFM; Invitrogen) with antibiotics as above. For RNA extraction, stromal and epithelial primary cell cultures were grown until 70% confluent and were lysed directly, without trypsinisation.

### RNA extraction and reverse transcription

Total RNA from the prostate tissues was isolated by first homogenising tissues in RNAzol (Biogenesis, Poole, UK) and then by using the Promega SV Total RNA Isolation System (Promega Corporation, USA) to remove DNA and purify the RNA. RNA was resuspended in nuclease free water and concentrations determined by spectrophotometry using a GeneQuant *pro* RNA/DNA calculator (Amersham Pharmacia Biotech, Buckinghamshire, UK). For the primary cell cultures, total RNA was extracted using an RNeasy mini kit (Qiagen). In total, 1 *μ*g of total RNA was reverse transcribed using 2 *μ*g random hexamers (Amersham) and 200 U of Superscript II reverse transcriptase (Life Technologies, Paisley, UK), according to the supplier's instructions. cDNA was stored at −20°C until used in the PCR reaction.

### Quantitative real-time PCR

For PCR reactions, specific primers and fluorogenic probes for all known human *MMP* and *TIMP* genes, *RECK, hepsin*, matriptase-1 (*MTSP1*), matriptase-2 (*MTSP2*), *maspin*, urokinase-type plasminogen activator (*uPA*) urokinase-type plasminogen activator receptor (*uPAR*), hepatocyte growth factor and activator (*HGF* and *HGFA*), and plasminogen activator inhibitor types-1 and -2 (*PAI1, PAI2*) were designed using Primer Express 1.0 software (PE Applied Biosystems) and synthesised by PE Applied Biosystems; sequences for primers and probes for the serine proteases are given in Table 1. Where possible, to prevent the amplification of genomic DNA and to ensure that the PCR signal was generated from cDNA, primers were generated with sequences within different exons, close to intron–exon boundaries. BLASTN searches (Altschul *et al*, 1990) were conducted on all primer/probe nucleotide sequences to ensure gene specificity. The identity of PCR products was confirmed by direct sequencing of the amplicon. The 18S ribosomal RNA gene was used as an endogenous control to normalise for differences in the amount of total RNA in each sample, using previously validated procedures (Wall and Edwards, 2002; Nuttall *et al*, 2003); 18S rRNA primers and probe were purchased from PE Applied Biosystems. PCR reactions were performed as described (Nuttall *et al*, 2003), with each reaction containing 5 ng of reverse transcribed RNA in 25 *μ*l. To determine the relative RNA levels within the samples, standard curves for the PCR reaction were prepared by using the cDNA from one sample and making two-fold serial dilutions covering the range equivalent to 20–0.625 ng of RNA (for 18S analyses, the range was 4–0.125 ng).

### Statistical analysis

The data did not satisfy normality or equal variance, so nonparametric tests were used. The Mann–Whitney *U* test was carried out to compare malignant and nonmalignant samples. Further tests were carried out using the two-tailed Spearman rank correlation coefficient to determine whether there were associations with the Gleason sum score. Finally, the epithelial and stromal cell culture populations derived from malignant prostate samples were also compared using the nonparametric Mann–Whitney *U*-test. For all tests, a *P*-value of less than 0.05 was considered statistically significant.

## RESULTS

### Expression of proteases and inhibitors in surgical specimens of prostate tissue

Our tissue banking technique and quality assurance procedures have been described in detail (Riddick *et al*, 2003). Briefly, a 9-mm punch was used to remove a cylindrical core of tissue from the transversely sliced prostate gland. The core was divided into four quadrants prior to banking by snap freezing in isopentane supercooled in liquid nitrogen. The histopathology of tissue at the circumference of the remaining prostate from which the punch was removed was used to predict the histopathology of the banked tissue as previously validated (Riddick *et al*, 2003). Our expression profiling strategy has focussed on secreted proteases and their inhibitors, including 28 genes that encompass the entire human *MMP* and *TIMP* families, as well as the novel MMP inhibitor *RECK*. In addition, we have assessed the expression of the components of the plasminogen activation system and the novel transmembrane serine proteases, *hepsin, MTSP1* and *MTSP2*, the inhibitors *PAI1, PAI2 HAI1, HAI2* and the serpin-related protein, *maspin*. The serine proteases, prostate-specific antigen (*PSA*)*, HGFA* and *tPA* were also included for the purpose of comparison.

**Figure 1:**
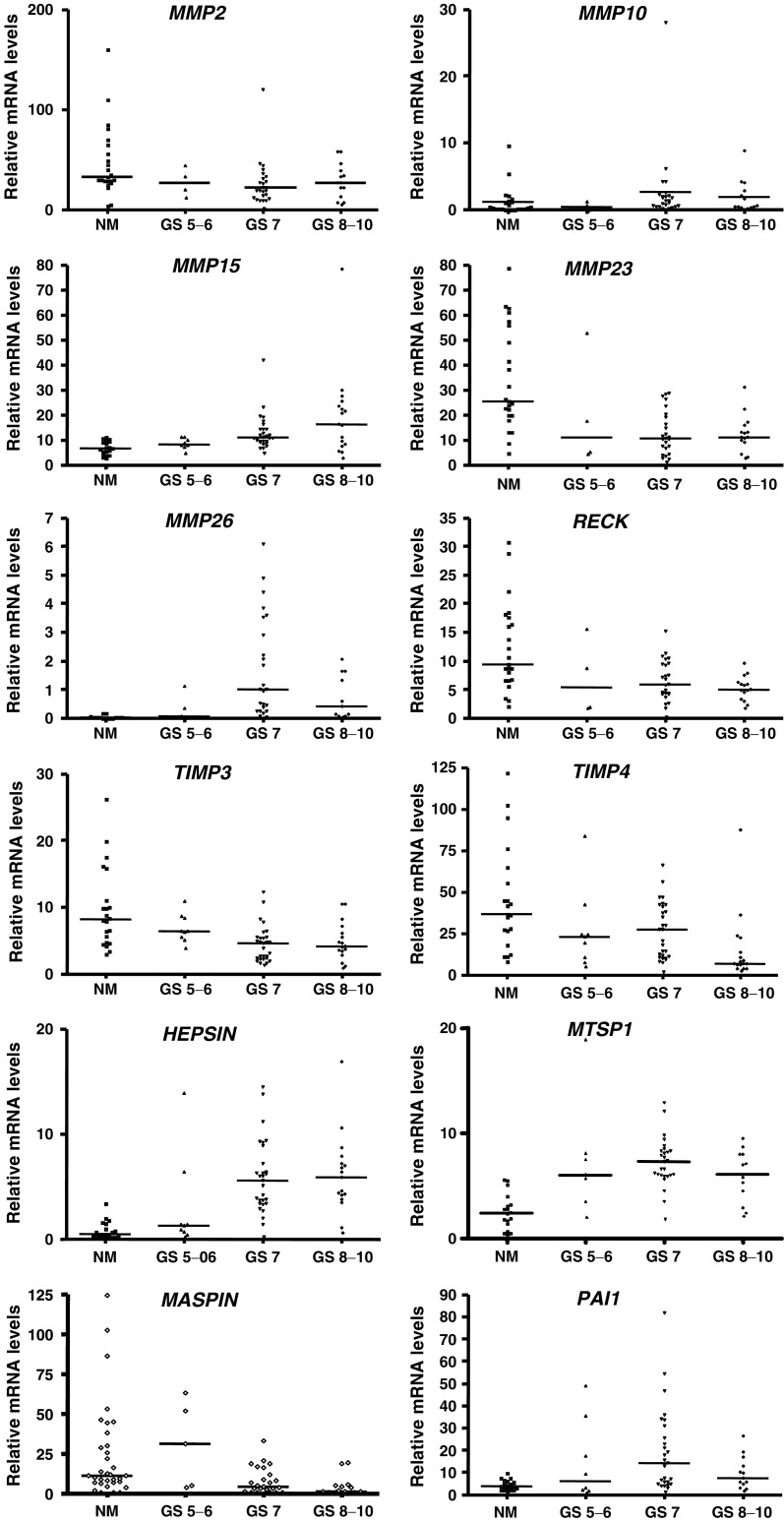
Quantitative RT–PCR analysis of selected genes showing differential expression in benign *vs* malignant prostate tissues. Prostate tissue specimens were sorted by Gleason Score (GS), and grouped as those with scores of 5–6, 7 or 8–10. The values of gene output are after normalisation to 18SrRNA and are probe, and therefore gene specific, thus precluding comparison of expression between genes. For summary of statistics see Table 2.

The complete data set of the RNA expression levels of protease and inhibitor genes in benign and malignant human prostate tissues is shown in Table 2 and data for selected genes are shown in Figure 1. An overall comparison of malignant prostate tissues with nonmalignant tissue samples obtained from prostates with BPH revealed that gene expression levels of several MMPs and serine proteases were significantly higher in the malignant samples. These proteases include *MMP10, 15, 24, 25* and *26, hepsin, MTSP1, uPAR* and also the inhibitor *PAI1*. By contrast, the levels of expression of *MMP2* and *MMP23* were significantly lower in the malignant samples, as were the inhibitors *TIMP3, TIMP4, RECK*, and the serpin-related gene *maspin*. No significant difference was seen between malignant and BPH samples for the expression of the remaining members of the MMP family.

The data were further analysed to correlate gene expression to increasing Gleason score (Gleason and Mellinger, 1974). Using the Spearman correlation coefficient, we identified statistically significant positive correlations with Gleason score for *MMP10, 15, 24 and 26*, and *hepsin, MTSP1*, *uPAR* and *PAI1* when comparing the increasing grades to benign or BPH controls (Table 2). In contrast, there were statistically significant negative correlations for *MMP2, MMP23, maspin, TIMP3, TIMP4* and *RECK*.

### Comparison of gene expression between primary stromal and epithelial cells cultured from malignant prostates

**Figure 2:**
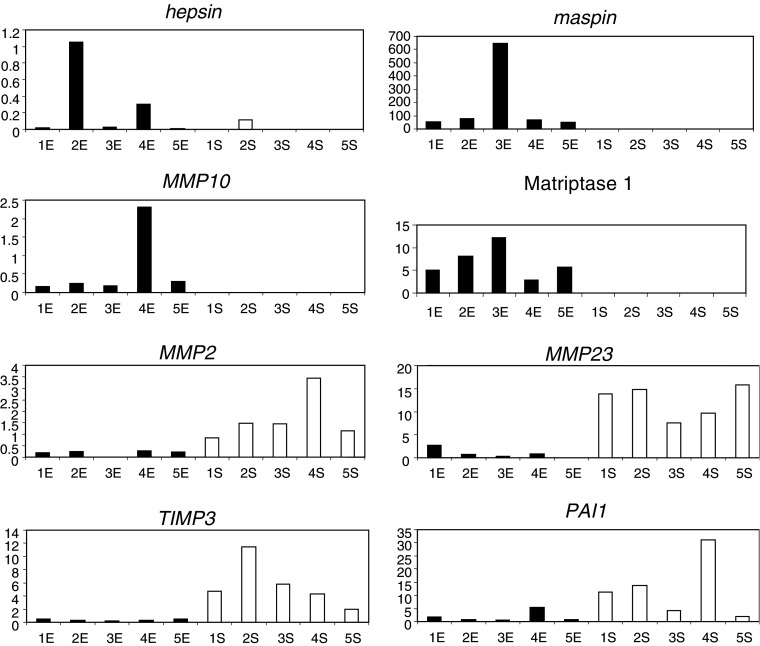
Differential expression of proteases in epithelium or stroma in primary prostate cell cultures. The figure shows QRT–PCR analysis of levels of expression of selected genes in epithelial (black columns) and stromal (white columns) primary cell cultures from human prostate tumours. Genes with a primarily epithelial pattern include *hepsin, maspin, MTSP1* (*matriptase-1*) and *MMP10*, whereas stromally expressed genes are *MMP2*, *MMP23*, *TIMP3* and *PAI1*. The values of gene output are after normalisation to 18SrRNA and are probe, and therefore gene specific, thus precluding comparison of expression between genes. For summary of statistics see Table 3.

Components of protease networks are frequently produced as a host stromal response to the presence of malignant tumour cells in human neoplasms. Thus, in order to define the cellular origins of the proteases identified from the comprehensive gene expression profile from bulk prostate tissue specimens, we undertook analysis of primary cultures of epithelial and stromal cells from radical prostatectomy tissues. Gene expression profiling of the primary malignant epithelial and stromal cell types showed the statistically significant and predominantly epithelial expression of *MMP10* and *25, hepsin, MTSP1* and *maspin* (Table 3; Figure 2). By contrast, there was a significantly greater expression of *TIMP3, TIMP4, RECK, MMP2*, and *23, uPAR* and *PAI1* in stromal cells compared to their epithelial counterparts. There were no significant differences in the levels of gene expression in the epithelial and stromal cells for *MMP15, 24* and *26*.

## DISCUSSION

This study is the first comprehensive analysis of the expression of the entire *MMP* and *TIMP* families in a large series of human prostate tissue samples, including both malignant (adenocarcinomatous) and nonmalignant (hyperplastic) tissue. In addition, the differential expression of members of the serine protease family emphasises the importance of evaluating more broadly the components of the ‘degradome’ – the repertoire of proteases, their inhibitors and associated molecules that each tissue deploys (Lopez-Otin and Overall, 2002). Our data identify several degradome genes as being differentially expressed, suggesting roles in prostate tumorigenesis or as markers of prognosis. The observations on fresh-frozen tissue samples have been extended by analysis of primary cell cultures of prostatic adenocarcinoma cells and stromal cells from the same neoplasms.

### Matrix metalloproteinases

Of the MMPs studied, the greatest increase (∼30 fold) in expression levels in malignant compared to nonmalignant prostate tissues was found for *MMP26*. The expression of *MMP26* was also found to correlate strongly with Gleason score, indicating its potential as a prognostic marker in prostate carcinoma. *MMP26* has only recently been implicated in tumorigenesis compared to some of the earlier discovered MMPs, and its expression has been found to be elevated in various epithelial neoplasms (Marchenko *et al*, 2001). Indeed, Zhao *et al* (2003) have demonstrated elevated expression in prostate cancer by immunohistochemical analysis of a limited number of human specimens. Their findings are consistent with the data presented in this study. The same authors also reveal a mechanism of action for MMP-26 by demonstrating its ability to activate pro-MMP-9 in ARCaP cells. In other studies, Zhao *et al* (2004) showed that MMP-26 is important in the activation of MMP-9 in breast carcinomas, where its activity is inhibited by TIMPs-2 and -4, suggesting that the interplay between these factors may be a significant determinant of malignancy in both breast and prostate neoplasia.

Two MMPs (*MMP2* and *MMP23*) were downregulated in prostate cancer. There is growing recognition that MMPs can exert inhibitory effects on tumour growth, potentially through the generation of angiogenesis inhibitors such as endostatin and tumstatin by cleavage of ECM components (Kalluri, 2003; Lafleur *et al*, 2003). Also, the absence of an MMP can promote tumorigenesis, as shown by the enhanced susceptibility to skin carcinogenesis displayed by *Mmp8*-null mice (Balbin *et al*, 2003). It is not clear at present whether *MMP2* and *23* might fulfill such a role in prostate tumorigenesis, though their downregulated expression suggests this as a possibility that warrants further study.

Our data for *MMP2* are consistent with those of Lichtinghagen *et al* (2002), who studied the expression of *MMP2* in human prostate tissues using quantitative RT–PCR, but differ from observations of MMP-2 protein levels using immunohistochemistry, where levels were increased in prostate cancer tissues (Brehmer *et al*, 2003), and from a study that identified an increase in transcript level in the highest grades of cancer (Still *et al*, 2000). Elevated plasma MMP-2 activity has also been suggested to be prognostic for prostate cancer metastasis (Morgia *et al*, 2005), indicating that there may be a discrepancy between *MMP2* RNA and protein levels in prostate tumours. However, the precise spatial expression of *MMP2* and its activators and potential substrates will determine its role in disease pathogenesis, and are likely be of more significance than its global level of expression.

To date, there are no data concerning expression of membrane-bound MMP-23 at the protein level. Elevation of three membrane-type-MMPs (MT-MMPs) was also demonstrated in this study, namely *MMP15, MMP24* and *MMP25* (*MT2-, MT5-* and *MT6-MMPs*). These MT-MMPs can post-translationally activate pro-MMP-2 (Llano *et al*, 1999; Nie and Pei, 2003). They also display intrinsic proteolytic activity towards ECM molecules, which is independent of MMP-2 activation. These proteases could thus participate directly in prostate tumour invasion, and in this regard it is important to note that *MMP25* (*MT6-MMP*) is primarily expressed by the epithelial cancer cells rather than stromal cells. This was also the case for *MMP10*, which likewise showed elevated expression in prostate cancer epithelium. Of note, Ernst *et al* (2002) found no difference in expression of *MMP10* between total biopsies of benign and malignant prostate tissue. However, comparison of microdissected malignant epithelium with benign epithelium revealed a marked elevation of *MMP10* in the malignant cell population. This may explain why we found a modest increase in *MMP10* in the malignant samples, but were unable to determine which Gleason grade was responsible for the increase. Other studies have observed *MMP10* upregulation in oesophageal carcinomas (Mathew *et al*, 2002), recurrent lung carcinomas (Cho *et al*, 2004) and in gliomas (Thorns *et al*, 2003), where they predict a more malignant phenotype. Similarly, Van Themsche *et al* (2004) found that induction of *MMP10* expression in lymphoma cell lines promoted their malignant phenotype.

### TIMPs

The tissue inhibitors of metalloproteinases, which act as key regulators of the MMPs, have important roles in tumorigenesis. The expression of *TIMP3* and *TIMP4* decreased progressively with increasing Gleason score, supporting the classic notion that these inhibitors may be protective in tumour progression. Therefore, reduced levels of expression of *TIMP3* in this study verifies the findings of Stamey *et al* (2001), who identified reduced levels of *TIMP3* expression by microarray analysis (in prostate tumour samples compared with benign prostatic hyperplasia) and those of Yamanaka *et al* (2003) who found hypermethylation of the *TIMP3* gene promoter (resulting in repressed expression). The mechanisms of action for the TIMPs vary between members, with some displaying growth promoting and antiapoptotic effects in some tumour contexts. In addition, there is evidence for effects that are independent of MMP inhibition. TIMP-3, which emerges as one of the most consistently tumour inhibitory TIMPs, can promote apoptosis by its selective effects on A Disintegrin And Metalloproteinase (ADAM)-mediated shedding of death-domain containing receptors (Ahonen *et al*, 2003). Independent of its MMP inhibitory activity, TIMP-3 suppresses angiogenesis by blocking the interaction of VEGF with VEGFR-2 (Qi *et al*, 2003). On the other hand, TIMP-4 can either promote (Jiang *et al*, 2001; Tunuguntla *et al*, 2003) or inhibit tumour progression, depending on the nature of the tumour and mode of delivery (Wang *et al*, 1997; Celiker *et al*, 2001; Groft *et al*, 2001; Zhao *et al*, 2004).

*TIMP1* and *TIMP2* expression on the other hand was not altered in our study. There have been previous studies demonstrating both elevated (Hashimoto *et al*, 1998) and reduced levels of expression (Lokeshwar *et al*, 1993; Brehmer *et al*, 2003) of these genes using semiquantitative methods of analysing gene expression. Clearly, further studies are required, particularly in view of the emerging theme of TIMP-1 supporting tumour progression in lymphomas (Guedez *et al*, 1998; Kossakowska *et al*, 2000; Oelmann *et al*, 2002; Kuittinen *et al*, 2003; Liu *et al*, 2003), colorectal carcinoma (Holten-Andersen *et al*, 2005), carcinoma of the breast (Lee *et al*, 2003; Porter *et al*, 2004), and skin tumorigenesis (Rhee *et al*, 2004). RECK, a novel metalloprotease inhibitor, is tentatively thought to limit tumour progression by inhibition of MMPs-2, -9 and -14 (each of which affects angiogenesis). Downregulation of *RECK* was seen with increasing Gleason score, similar to *TIMP3* and *TIMP4*. Notably, *TIMP3, TIMP4* and *RECK* were all expressed predominantly by stromal cells. We considered whether the decreased expression of these stromally expressed genes (and also *MMPs-2* and -*23*) in cancers compared to benign tissues might reflect a change in the proportion of epithelial to stromal cells due to expansion of the malignant epithelial compartment. However, other genes that show a stromal expression pattern did not show the same reduction, for instance, *PAI1* and *uPAR* are elevated (see below) and *HGF*, also stromal in origin (data not shown) was unaltered in overall expression between benign and malignant tissues. Thus, we argue that the proportion of stromal cells in the prostate tissue homogenates does not vary appreciably between benign and malignant tissues, and therefore the reduced expression of *TIMP3, TIMP4* and *RECK* is most likely a functional consequence of tumour–stromal interactions.

### Serine proteases and inhibitors

Serine proteases of the uPA/plasminogen/plasmin cascade have been implicated in prostate cancer progression, where they may act independently or may activate MMPs. Our data show elevated transcript levels of *PAI1* and *uPAR* in cancer compared to benign tissues, with no significant changes in expression of *uPA* mRNA levels. Interestingly soluble uPAR (suPAR) levels are elevated in the serum of prostate cancer patients (Miyake *et al*, 1999; McCabe *et al*, 2000). It is likely therefore that the increased *uPAR* mRNA expression that we observe in prostate stromal cells at least partially underpins these increases. As with the TIMPs, the role of PAI1 in malignancy may be paradoxical, since in some models it promotes tumour invasion and angiogenesis. Moreover, elevated expression of *PAI1* has also been seen in lung, breast and ovarian carcinomas (Robert *et al*, 1999; Pedersen *et al*, 2000; Salden *et al*, 2000; Borgfeldt *et al*, 2001). PAI1 has effects both on cell adhesion and plasmin activation (Czekay and Loskutoff, 2004), and thus both the extent and location of its expression in relation to its interaction partners will determine its net contribution to malignancy. Ours are the first data to demonstrate elevated expression of *PAI1* in prostate cancer, and given current knowledge of the biology of PAI1, it is likely that it is relevant for the promotion of cell invasion or tumour vascularisation.

Our data identify the transmembrane serine proteases *hepsin* and matriptase-1 (*MTSP1*) as potentially strong prognostic markers of prostate cancer. The marked elevation of hepsin in malignant prostate tissues and correlation with increasing Gleason score were in agreement with other data from microarray analyses and real-time PCR studies (Dhanasekaran *et al*, 2001; Stamey *et al*, 2001; Ernst *et al*, 2002; Chen *et al*, 2003; Riddick *et al*, 2003; Stephan *et al*, 2004). The functional importance of hepsin has been shown by its overexpression in a transgenic mouse model of prostate tumorigenesis, which led to basement membrane disorganisation and enhanced metastasis (Klezovitch *et al*, 2004). Elevated expression of *MTSP1* in prostate cancer tissues and positive correlation with increasing Gleason score are also novel findings. The cell culture data indicate that its expression is predominantly epithelial in origin, which is in keeping with other tumour systems such as lung mesotheliomas (Hoang *et al*, 2004), ovarian (Oberst *et al*, 2002), breast (Lin *et al*, 1997; Takeuchi *et al*, 1999; Oberst *et al*, 2001) and in epithelial tissues in general (Oberst *et al*, 2003). Recent data have indicated that matriptase may have a role in the activation of hepatocyte growth factor (Lee *et al*, 2000) and it will be of interest to determine whether this is integral to its action in the prostate, where there is evidence for participation of increased HGF-Met signalling in tumour progression (You *et al*, 2003; Hall *et al*, 2004). Met and matriptase are epithelial in origin, whereas HGF is stromally derived, thus supporting the importance of epithelial–stroma interaction in tumorigenesis (Lee *et al*, 2000).

Maspin, which has been shown to have tumour-suppressive effects, was found to be downregulated in prostate cancer tissues in agreement with Chen *et al* (2003). Members of our group have shown that maspin is not functional as a serine protease inhibitor (Bass *et al*, 2002), which has now been confirmed at the structural level (Al Ayyoubi *et al*, 2004), where it has been shown that the reactive centre loop necessary for inhibitory function is maintained in a highly constrained, inactive conformation. Its tumour suppressive effects are likely due to other mechanisms, including promotion of cell adhesive mechanisms, as shown by Abraham *et al* (2003) where TRAMP C2N cells stably transfected with maspin exhibited a lower tumorigenicity demonstrated by a reduced growth rate, decreased metastatic potential and increased adhesiveness to fibronectin and laminin. In another model, Cher *et al* (2003) found that maspin inhibits osteolysis, tumour growth and angiogenesis.

## CONCLUSION

Our comprehensive survey has identified components of the degradome as potential markers of prostate carcinoma. These data identify *MMP2, MMP10, MMP15, MMP26, MMP23, TIMP3, TIMP4*, *hepsin, MTSP1, PAI1, uPAR* and *maspin* as genes that require further study at the protein level and via immunohistochemical analysis to elucidate their contributions to prostate cancer progression. The metalloproteinase and serine proteinase pathways are not independent of each other and, indeed, converge at the pericellular environment to mediate the proteolysis, which potentially facilitates invasion of the ECM and other structures by cancer cells. This study points towards future research avenues to elucidate these interactions and to establish their relevance in human prostate cancer.

## Figures and Tables

**Table 1:** Primer-probe sequences of the genes analysed by quantitative RT–PCR. Sequences for the primers and probes are shown in 5′ to 3′ orientation

**Gene name**	**Primer/probe sequence**
*Hepsin*	
Forward primer	GAGGAGAACAGCAACGATATTGC
Reverse primer	CCCGTCACGGTACAGATCTTG
Probe	CTCACAGAATACATCCAGCCTGTGTGCCT
	
*MTSP1*	
Forward primer	CACCTCAGTGGTGGCTTTCC
Reverse primer	GCGTGCAGGCCAAAGCT
Probe	CAAAACAGTACAGAGGACCCAGGACAACAGC
	
*MTSP2*	
Forward primer	CAGAAGATGCTCAAGGAGCTCAT
Reverse primer	GGAATAGACGGAGCTGGAGTTG
Probe	ACCAGCACCCGCCTGGGAACTTACT
	
*Maspin*	
Forward primer	CAGATGGCCACTTTGAGAACATT
Reverse primer	GGCAGCATTAACCACAAGGATT
Probe	GGCAGCATTAACCACAAGGATT
	
*UPA*	
Forward primer	TGGAACTCTGCCACTGTCCTT
Reverse primer	GGCCCACCTGCACATAGC
Probe	ATAATTACTGCAGGAACCCAGACAACCGGA
	
*UPAR*	
Forward primer	GCCCAATCCTGGAGCTTGA
Reverse primer	TTCCCCTTGCAGCTGTAACAC
Probe	CTGCCGCAGAATGGCCGCC
	
*PAI1*	
Forward primer	CAGAGGTGGAGAGAGCCAGATT
Reverse primer	CTGGTCCACGGCTCCTTTC
Probe	GTGAAGACACACACAAAAGGTATGATCAGCAACTT
	
*PAI2*	
Forward primer	AGTGTCAATAAGCTGTTTGGTGAGA
Reverse primer	TCTAGCTTCTTCTGCACATTCTAGGA
Probe	CTTCCGGGAAGAATATATTCGACTCTGTCAGAAA
	
*TPA*	
Forward primer	AAACCCAGATCGAGACTCAAAGC
Reverse primer	GGCTGACCCATTCCCAAAG
Probe	CCTGCCTGCTCTGAGGGAAACAGTGAC
	
*PSA*	
Forward primer	TCTGCGGCGGTGTTCTG
Reverse primer	CTGTGCCGACCCAGCAA
Probe	CCACTGCATCAGGAACAAAAGCGTGAT
	
*HGFA*	
Forward primer	CATCGAGAAGTACATCCCGTACAC
Reverse primer	CAGATGGGCTGCACGAACT
Probe	CCAGCGACCACGACCTCGTCCT
	
*HAI1*	
Forward primer	CGCGGCATCTCCAAGAAG
Reverse primer	AGCCTGTGCTGGGAATGG
Probe	TGTGTTTGGCCTGAGGCGGGA
	
*HAI2*	
Forward primer	GCTCTGAGGAGGCCTGCAT
Reverse primer	AAGAGGATCAACACCATCACGAA
Probe	CTCCGCTGCTTCCGCCAGCA

Probes contain an FAM fluorescent reporter on the 5′ end and a TAMRA quencher on the 3′ end. Primer-probe combinations for TIMP and RECK genes have been described elsewhere (Nuttall *et al*, 2003). For the MMPs and TIMPs, primer and probe sequences are the property of Applied Biosystems (Warrington, UK). HGFA=HGF activator.

**Table 2:** Summary of expression profiling of genes showing differential expression between benign and malignant tissues, and relationships with Gleason score

**Gene**	**Malignant *vs* non-malignant**	**Correlation with Gleason score**	**Direction of change in malignancy**
*MMP1*	*P*=0.911	*P*=0.533	
***MMP2***	***P*=0.004**	***P*=0.028/*R*=−0.286**	↓
*MMP3*	*P*=0.835	ND	
*MMP7*	*P*=0.106	NS	
*MMP8*	N/D	ND	
*MMP9*	*P*=0.501	ND	
***MMP10***	***P*=0.016**	***P*=0.015/*R*=−0.297**	↑
*MMP11*	*P*=0.539	ND	
*MMP12*	*P*=0.883	ND	
*MMP13*	N/D	ND	
*MMP14*	NS	NS	
***MMP15***	***P*<0.0001**	***P*<0.0001/*R*=0.484**	↑
*MMP16*	NS	NS	
*MMP17*	NS	NS	
*MMP19*	*P*=0.932	ND	
*MMP20*	N/D	ND	
*MMP21*	*P*=0.288	ND	
***MMP23***	***P*<0.0001**	***P*<0.0001/*R*=−0.497**	↓
***MMP24***	***P*=0.014**	***P*=0.011/*R*=0.289**	↑
***MMP25***	***P*=0.003**	**NS**	↑
***MMP26***	***P*<0.0001**	***P*<0.0001/*R*=0.507**	↑
*MMP27*	N/D	ND	
*MMP28*	*P*=0.736	ND	
			
*TIMP1*	*P*=0.183	ND	
*TIMP2*	*P*=0.126	ND	
***TIMP3***	***P*=0.001**	***P*<0.0001/*R*=−0.430**	↓
***TIMP4***	***P*=0.004**	***P*<0.0001/*R*=−0.411**	↓
***RECK***	***P*<0.0001**	***P*<0.0001/*R*=−0.423**	↓
			
***Hepsin***	***P*<0.0001**	***P*<0.0001/*R*=0.677**	↑
***MTSP1***	***P*<0.0001**	***P*<0.0001/*R*=0.496**	↑
*MTSP2*	*P*=0.052	NS	
*PSA*	*P*=0.702	NS	
*HGFA*	*P*=0.424	NS	
*TPA*	*P*=0.054	NS	
***uPA***	***P*=0.04**	***P*=0.706/*R*=0.047**	↑
***uPAR***	***P*=0.42**	***P*=0.014/*R*=0.290**	↑
			
***PAI1***	***P*=0.001**	***P*=0.004/*R*=0.35**	↑
***PAI2***	***P*=0.032**	***P*=0.028/*R*=−0.253**	↓
***Maspin***	***P*<0.001**	***P*<0.0001/*R*=−0.455**	↓
*HAI1*	*P*=0.076	NS	
***HAI2***	***P*=0.166**	***P*=0.015/*R*=−0.279**	↓

Arrows in the right-hand column illustrate direction of change in gene expression with malignancy. N/D=not detected, NS=not significant, ND=not done. The values given are derived from TaqMan expression data after normalisation to 18SrRNA levels, and are probe and, therefore, gene-specific. Comparison of expression between genes is therefore not possible. The statistical test used was the Mann–Whittney *U* test to compare malignant and non-malignant samples and the two-tailed Spearman rank correlation test. HGFA=HGF activator. Genes showing statistically significant differences are indicated in bold.

**Table 3:** Analysis of gene expression in primary epithelial and stromal fibroblast cell cultures from prostate tumours

**Gene**	**Epithelial**	**Stromal**	***P*-value**
*Gene expressed predominantly by malignant epithelial cell cultures*			
* MMP 10*	1	0.01	0.009
* MT6MMP*	1	0.09	0.009
* Hepsin*	1	0	0.04
* MTSP1*	1	0.01	0.008
* Maspin*	1	0	0.009
* cMet*	1	0.28	0.029
* HAI1*	1	0.05	0.029
* HAI2*	1	0.03	0.029
			
*Gene expressed predominantly by stromal cell cultures*			
* MMP2*	1	6.49	0.009
* MMP23*	1	21.44	0.009
* TIMP3*	1	15.22	0.009
* TIMP4*	1	7.6	0.047
* RECK*	1	50	0.009
* UPAR*	1	5.28	0.016
* PAI1*	1	14.37	0.028
* HGF*	1	1425	0.029
			
*Genes showing no significant difference in expression between cell cultures*			
* MMP26*	0.566	0.25	0.175
* MMP15*	1.071	0.702	0.602
* MMP24*	0.605	22.435	0.076
* uPA*	48.388	5.168	0.076
* HGFA*	1	1	1

The values given are derived from TaqMan expression data after normalisation to 18SrRNA levels, and are probe and, therefore, gene-specific. Comparison of expression between genes is not possible. All epithelial values have an arbitrarily fixed value of 1 and the stromal expression relative to this is shown. The statistical test used was the Mann–Whittney *U*-test.
